# Genetic constitution and variability in synthetic populations of intermediate wheatgrass, an outcrossing perennial grain crop

**DOI:** 10.1093/g3journal/jkae154

**Published:** 2024-07-13

**Authors:** Prabin Bajgain, Jacob M Jungers, James A Anderson

**Affiliations:** Department of Agronomy and Plant Genetics, University of Minnesota, 411 Borlaug Hall, 1991 Upper Buford Circle, St. Paul, MN 55108, USA; Department of Agronomy and Plant Genetics, University of Minnesota, 411 Borlaug Hall, 1991 Upper Buford Circle, St. Paul, MN 55108, USA; Department of Agronomy and Plant Genetics, University of Minnesota, 411 Borlaug Hall, 1991 Upper Buford Circle, St. Paul, MN 55108, USA

**Keywords:** perennial, synthetic, breeding, simulation, genomic prediction

## Abstract

Intermediate wheatgrass (IWG) is a perennial grass that produces nutritious grain while offering substantial ecosystem services. Commercial varieties of this crop are mostly synthetic panmictic populations that are developed by intermating a few selected individuals. As development and generation advancement of these synthetic populations is a multiyear process, earlier synthetic generations are tested by the breeders and subsequent generations are released to the growers. A comparison of generations within IWG synthetic cultivars is currently lacking. In this study, we used simulation models and genomic prediction to analyze population differences and trends of genetic variance in 4 synthetic generations of MN-Clearwater, a commercial cultivar released by the University of Minnesota. Little to no differences were observed among the 4 generations for population genetic, genetic kinship, and genome-wide marker relationships measured via linkage disequilibrium. A reduction in genetic variance was observed when 7 parents were used to generate synthetic populations while using 20 led to the best possible outcome in determining population variance. Genomic prediction of plant height, free threshing ability, seed mass, and grain yield among the 4 synthetic generations showed a few significant differences among the generations, yet the differences in values were negligible. Based on these observations, we make 2 major conclusions: (1) the earlier and latter synthetic generations of IWG are mostly similar to each other with minimal differences and (2) using 20 genotypes to create synthetic populations is recommended to sustain ample genetic variance and trait expression among all synthetic generations.

## Introduction

With an increasing degree of uncertainty in climatic patterns, it is more imperative to increase and stabilize crop yield per acre to produce enough nutritious food for the human population. The amount of arable land available to humans is not projected to grow, yet the demand for food, especially in the face of rising erratic environmental patterns, continues to grow (Maulu *et al.* 2021). It is also hypothesized that current agriculture practices need to be modified to limit negative environmental consequences and improve input use efficiency, and growing perennial plants is proposed as one potential solution (Leisner 2020).

Intermediate wheatgrass (IWG, *Thinopyrum intermedium*) is a perennial crop with an extensive root system, large biomass, and edible grain. IWG provides substantial ecosystem services such as reduced water and soil erosion, reduced nitrate leaching, and a lower need for resource inputs (Glover *et al.* 2010; Culman *et al.* 2013; Jungers *et al.* 2019). The IWG breeding and domestication project within the Perennial Cereals Breeding Program at the University of Minnesota (UMN) was initiated in 2011. IWG breeding at the UMN utilizes a recurrent selection scheme that combines conventional plant breeding techniques such as population assessment at multiple locations and years and modern genetic and genomic tools such as high-throughput genotyping and genomic selection (GS; Bajgain *et al.* 2022b).

The breeding pipeline can broadly be categorized into 2 main schemes: (1) population improvement and (2) variety development. The population improvement scheme focuses on germplasm enhancement through phenotypic evaluation, marker discovery, selection, and controlled crosses. The variety development scheme focuses on creation of synthetic (SYN) variety candidates, state-wide variety trials, seed increase, and public release of the best variety. Varieties could be sold as Kernza perennial grain, and Kernza is a trade name for food-grade IWG grain owned by The Land Institute, Salina, KS, USA (DeHaan and Ismail 2017). In particular, crossing blocks are initiated by planting several, replicated clones of genotypes that performed the best in field trials for 2–3 years (Bajgain *et al.* 2020b). Plants are typically spaced at 1 m on a 7 × 7 grid (area of 36 m^2^). The blocks are also physically isolated by a distance of 15 m (50 ft) that is interspaced by tall, winter rye varieties to minimize pollen travel among the blocks. The crossing block is called the “SYN0” generation to represent the “zero harvest generation.” Subsequent seed increases are carried out in isolated plots in different field locations and are designated SYN1, SYN2, SYN3, and so on with each year of seed increase or generation advancement. In other words, these SYN populations represent panmictic multiplication generations. Variety trials are carried out with a SYN2 generation, and the commercially released variety is typically a SYN4 generation.

In the recent decades, plant breeding is being revolutionized by the availability of highly affordable genome-wide polymorphic markers, primarily SNPs, because of the advances made in improving high-throughput genotyping methods. Breeding methods are also becoming more efficient, e.g. partly through adoption of robust statistical models that enable fast and accurate gene mapping and trait predictions (Bradshaw 2017; Kuriakose *et al.* 2020). A high-quality draft of the IWG genome assembly is also complete and is available to researchers as part of their analytical toolbox [*Thinopyrum intermedium* (2024) Genome Sequencing Consortium]. The draft genome, together with the availability of less-expensive sequencing technologies and efficient bioinformatic and statistical algorithms, has made it possible to discover genomic markers. In our previous research work in IWG, we have used SNP markers to conduct studies spanning from gene discovery (Bajgain *et al.* 2022c), determine pollination distance (Bajgain *et al.* 2022a), and evaluate the efficacy of using genotype by environment (G × E) interaction in conducting genome-wide selection work in an applied breeding program (Bajgain *et al.* 2020a, 2022b). For a perennial species with no other form of genotyping, e.g. marker panel such as SNP-chips and PCR-based markers, the ease of carrying out high-throughput sequencing to obtain thousands of informative genomic markers has been immensely helpful in improving the rate of domestication for this new perennial crop.

Current IWG breeding programs routinely collect genome-wide marker information and phenotype data to improve traits such as seed shattering, free grain threshing, seed size, grain yield, and disease resistance. In the UMN IWG breeding program, evaluation nurseries that comprise several hundred individual IWG genotypes are established at UMN's Southwest Research and Outreach Center in Lamberton and in St. Paul and are maintained for 2 years. Collected phenotype and genotype data are combined to map genes and train GS models. Afterwards, the GS model is applied to a larger, untested population to predict its performance and select the best individuals for the next breeding cycle. A subset of the best individuals are also planted in crossing blocks to develop SYN varieties, as described above. The GS models we implement are updated regularly and sometimes changed in favor of new, more effective models to boost prediction accuracies. For example, we have started using models that utilize G × E effects as these models improved trait predictions by 18%, 15%, 20%, and 23% for grain yield, spike weight, spike length, and free threshing, respectively (Bajgain *et al.* 2020a). As GS plays a pivotal role in making breeding IWG more efficient and shortening IWG's breeding cycle, continual optimization of GS models is necessary to increase IWG's domestication rate and develop better varieties.

**Fig. 1.**
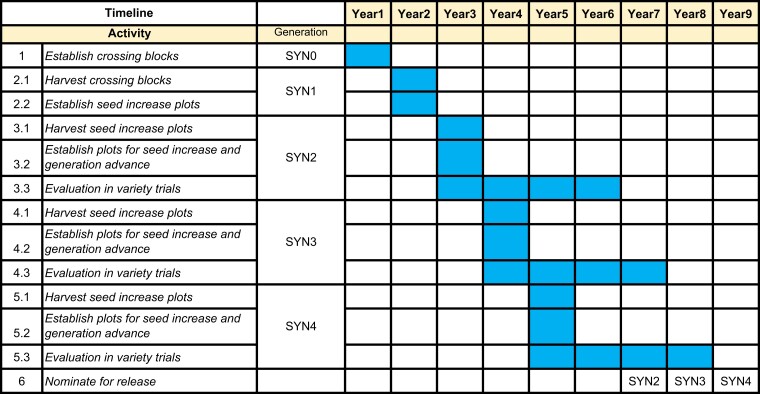
Variety development timeline to create IWG SYN populations at the UMN, conduct variety trials, and recommend a candidate to be released as a variety.

Despite the progress in trait improvement and population development, perennials require more time to develop, test, and release to farmers compared with annuals (Fig. 1). In UMN's IWG breeding program, selection is carried out in spaced plants that do not mimic a farmer's field where plants are established in rows or swards. The SYN populations of IWG varieties are developed by crossing individuals selected from a concurrent breeding cycle, advanced for 4–5 generations to obtain enough seed for variety testing and eventual public release as a variety. During this step, grain from different SYN generations is sourced to conduct different research experiments. As a wind-pollinated outcrossing perennial species, IWG's genetic composition changes each time the plants are allowed to open pollinate. Despite different SYN generations being used in yield trials and variety release, the performance differences among different SYN generations are yet to be studied. It is not understood if there are significant differences in genetic composition and variability in earlier SYN generations that are used by breeders, agronomists, and other researchers compared with subsequent SYN generations that are eventually made available to growers as varieties. Releasing the variety with data obtained from an earlier generation (SYN2) also means that farmers would get access to the variety sooner and allow breeding programs to save resources by not having to increase and evaluate more generations.

With these considerations, we conducted this study to evaluate the differences among IWG SYN generations. We did this by characterizing population diversity, genetic differences, and trait performances through the application of simulation as well as genomic prediction models. The simulation models use true genome-wide markers obtained from our first breeding cycle, which was used to estimate additive genetic variance for the populations. We also used the simulation models to identify the optimal number of parental genotypes to be used to develop IWG SYN varieties. The results provide valuable insights on differences among the SYN generations of IWG which can be followed by breeders to develop future varieties.

## Materials and methods

### Plant population

This study evaluated 4 multigenerational IWG populations of the commercially released cultivar MN-Clearwater. Each generation is a SYN population obtained by open pollinating the previous generation without any selection imposed on the plants, and the process of SYN development and timeline is described in detail by Bajgain *et al*. (2020b). Briefly, to obtain the first SYN generation SYN1, a crossing block (often referred to as the SYN0 generation) was established by transplanting 49 plants (7 unique genotypes replicated 7 times) at UMN's Sand Plain Research Farm in Becker, MN. The SYN1 grain harvested from Becker was planted in another isolated field in Becker to obtain SYN2 seeds. Likewise, SYN3 was obtained by planting SYN2 seed from Becker in a farmer's field in Roseau, MN; and SYN4 was obtained from planting SYN3 seed obtained from Roseau in UMN's Rosemount Research and Outreach Center in Rosemount, MN. All fields were fertilized with approximately 50 kg ha^−1^ of N (urea) in the spring (April to May) of each year. Sizes of the populations used in this study were 437, 250, 192, and 194 genotypes for SYN1, SYN2, SYN3, and SYN4, respectively.

### Genotyping

Grains from all populations were germinated in a professional soil mix at a UMN plant growth facility during September to October 2020. A random mix of seed from each generation was sown in the soil mix, and 10–12 cm of leaf tissue from the seedlings was collected for DNA extraction. Tissue was dried on a bed of silica for 4 days, and DNA was extracted using the BioSprint 96 DNA Plant Kit (QIAGEN, Valencia, CA). Population genotyping was accomplished by following the genotyping by sequencing method (Poland *et al.* 2012) on Illumina Novaseq 6,000 at the University of Minnesota Genomics Center with a target of 1 million reads per sample. Obtained reads were quality filtered (Q > 30) and aligned to the *Thinopyrum intermedium* v3.1 reference genome [*Thinopyrum intermedium* (2024) Genome Sequencing Consortium] using bwa 0.7.5a (Li 2013). Programs SAMtools 1.6 and BCFtools 1.6 (Li *et al.* 2009; Li 2011) were used with default parameters for detection of genome-wide SNPs. The SNPs were filtered for a minimum minor allele frequency of 3%, and variants with missing proportion of >10% were removed. Genotypes with less than 80% of the alleles called were discarded. These steps resulted in 13,633 SNP markers which were imputed using the LD-kNNi method (Money *et al.* 2015) using Tassel 5.2.70 (Bradbury *et al.* 2007). The imputed data set was used in all downstream analyses.

### Population properties

Changes in linkage disequilibrium (LD) in different SYN generations of MN-Clearwater were also estimated using Tassel 5.2.70. Evaluation of existent population structure among the generations was done using principal component (PC) analysis in R 4.2.3 with the function “prcomp” (R Core Team 2023). To further investigate the relationship among the 4 SYN generations, measures of genetic differentiation and population diversity were carried out. These measures included the Weir and Cockerham fixation index (Fst; Weir and Cockerham 1984), Nei's genetic distance (*D*; Nei 1972), and Shannon's diversity index (*H*; Hill 1973). Estimates of these diversity measures were done in R 4.2.3: Fst using the package “snpStats,” *D* using “StAMPP” (Pembleton *et al.* 2013), and *H* using “vegan” (Oksanen *et al.* 2020).

### Population simulation

In the UMN IWG breeding program, a regular and detailed phenotyping of the SYN populations is rarely done. The only exception is the SYN2 generation, as this is the generation used in replicated performance testing and is also the source of breeder's seed. Rather, spaced plants are evaluated for several agronomic traits such as grain yield, plant height, and disease resistance and domestication traits such as shatter resistance, free grain threshing, and seed size. The best spaced plants are then selected based on their field performance and crossed in isolation to obtain the SYN1 generation. As no trait data exist for the SYN generations used in this study, we relied on a simulation algorithm to obtain genetic makeup of these SYNs and their phenotypic values and variances. The base or starting population is the SYN0 population with known genotypic data of the parent plants from the Cycle 1 IWG breeding population, from which the parents of MN-Clearwater were selected. We also informed the model of the trait architecture in IWG based on research results reported by previous studies (see review article by Bajgain *et al.* 2022b). All simulations were carried out using the “AlphaSimR” package (Gaynor *et al.* 2021).

In the first step, 5 SYN1 populations were initiated from 7, 10, 15, 20, and 25 parents. For all 5 populations, SYN2–SYN4 generations were advanced from their respective SYN1 generations without imposing any selection at any step. This was done to assess any effect on trait variance of a population initiated with varying number of parents and to determine an optimal number of parents to be used in initiating SYN populations. A polygenic trait architecture was simulated with number of genome-wide causative loci (i.e. QTL) set at 1,000, and trait heritability (*H*^2^) value of 0.6 was used. An overall trait mean of 1 was used, and trait mean is defined by the AlphaSimR package as the “vector of desired mean genetic values for one or more traits.” We also assumed that the additive variance (σ^2^_a_) equals the overall genetic variance (σ^2^_g_) and other variances, i.e. dominance, G × E interaction, and residual, were assumed to be absent. Simulating a large population that accurately resembles the IWG populations in our fields (several hundred thousand plants) was computationally restrictive. The number of progeny per SYN generation was therefore set to 100,000 from 1,000 random crosses with 100 progeny per cross. In the second step, simulation was repeated with 2 additional *H*^2^ estimates: 0.4 and 0.8, in addition to *H*^2^*=* 0.6. In both steps, simulation models were run for 100 iterations. Significant differences in group means among the SYN generations and different *H*^2^ levels were determined using Tukey's honestly significant difference (HSD) test at *α* = 0.001 level using the R package “agricolae” (de Mendiburu 2020).

### Genomic trait prediction

In addition to simulating SYN populations and their trait information, trait values for SYN1–SYN4 were also obtained from genomic prediction. Phenotypic data for the SYN0 plants of MN-Clearwater were collected from our Cycle 1 breeding nursery. These data were obtained during 2012–2013 from St. Paul, MN, and have been explained in a previous study (Zhang *et al.* 2016). Trait data from Cycle 1 breeding population were therefore used to predict trait values of the genotyped individuals of SYN1–SYN4 individuals. Prediction was carried out using a ridge regression model of the following nature:


y=Xβ+Zu+ε

where *y* is a vector of *N*_TP_ × 1 of field-observed phenotypes for each plant in the Cycle 1 training population, *X* is a *N*_TP_ × *N*_Yr_ design matrix of the Cycle 1 training population evaluated for 2 years, β is a *N*_Yr_ × 1 vector of fixed effects of years in multiyear evaluation of Cycle 1 training population, *Z* is a *N*_TP_ × *N*_geno_ design matrix relating *y* to *u*, *u* is a *N*_geno_ × 1 vector of random marker effects, ε is a vector of *N*_TP_ × 1 residuals, and *N*_TP_, *N*_Yr_, and *N*_geno_ are the training population size, number of evaluation years, and number of SNPs in the genotype matrix, respectively.

Predictions were carried out using the model as implemented in the “rrBLUP” package (Endelman 2011), and a 4-fold cross validation method was used to assess model's ability to predict the traits. In the 4-fold cross validation method, 75% of the population size for each generation was used as training set and the remaining 25% was used as the validation set. Mean trait values were obtained by averaging 100 model replications. Significant differences in group means were determined using Tukey's HSD test at *α* = 0.001 level using the R package “agricolae” (de Mendiburu 2020).

## Results

### Genotyping and population diversity

Filtering of SNP markers obtained from genotyping by sequencing resulted in 13,633 genome-wide SNPs. The average marker distribution was 649 SNPs per chromosome (range of 521–855) with genome-wide average minor allele frequency of 22%. Table 1 summarizes population diversity metrics such as Weir and Cockerham fixation index (Fst), Nei's genetic distance (*D*), and Shannon's diversity index (*H*). The SYN2 population had the lowest average Fst value (0.03), and SYN1 had the highest (0.07) with SYN3 and SYN4 having the same value of 0.05. Measures of Nei's *D* and Shannon's diversity index among the 4 populations were near identical with no significant differences.

**Table 1.** Parameters of population genetic diversity in 4 SYN generations of the IWG cultivar MN-Clearwater.

Measure	Generation	*N*	Min	Max	Mean
Fst	SYN1	437	0.01	0.77	0.07
	SYN2	250	−0.12	0.67	0.03
	SYN3	192	0.01	0.74	0.05
	SYN4	194	−0.09	0.70	0.05
*D*	SYN1	437	0.00	0.03	0.00
	SYN2	250	0.00	0.03	0.01
	SYN3	192	0.00	0.01	0.00
	SYN4	194	0.00	0.03	0.01
*H*	SYN1	437	9.50	9.52	9.51
	SYN2	250	9.51	9.52	9.51
	SYN3	192	9.48	9.52	9.51
	SYN4	194	9.51	9.52	9.51

Fst, mean Weir and Cockerham fixation index; *D*, Nei's genetic distance; *H*, Shannon's diversity index; *N*, population size.

### Population structure and LD

**Fig. 2.**
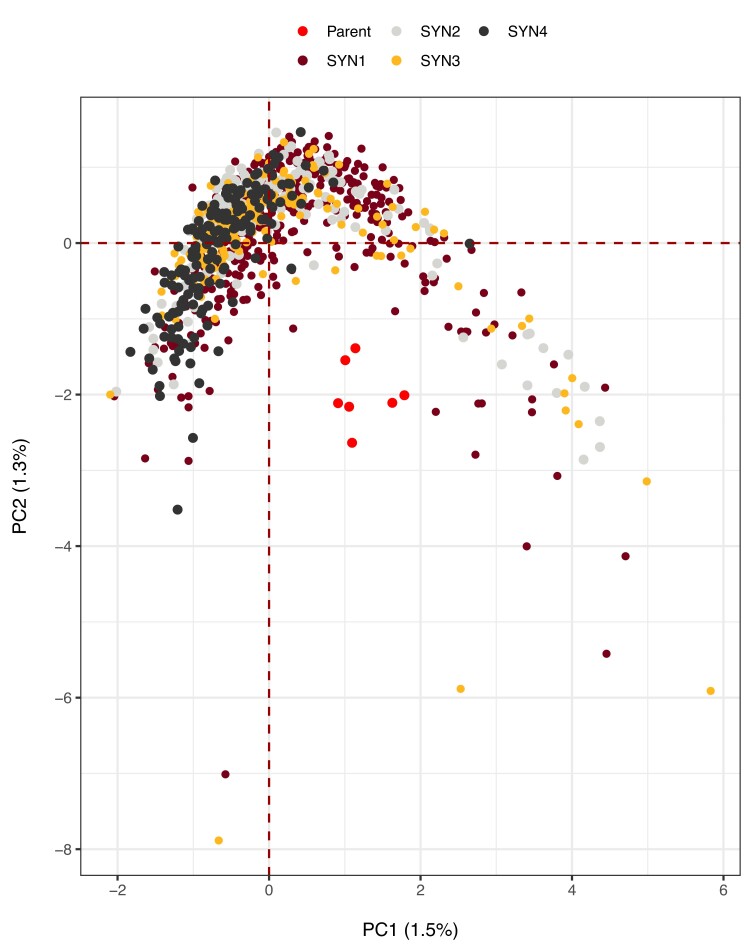
Population structure visualized using PC analysis among the 4 different SYN generations of the IWG cultivar MN-Clearwater. “Parent” indicates the 7 parents used to create MN-Clearwater.

Population substructures evaluated using PC analysis showed a weak structure (Fig. 2). The first 2 PC axes explained 2.8% of the genotypic variation and the first 10 axes explained only 7.1%. Most likely from the lack of a strong structure, a vast majority of the individuals from all SYN generations are clustered closely. In addition, nearly all individuals of all 4 SYN generations are clustered slightly away from the 5 parental genotypes. A few individuals in SYN1 and SYN3 populations were observed to be positioned differently from the rest of their respective clusters.

**Fig. 3.**
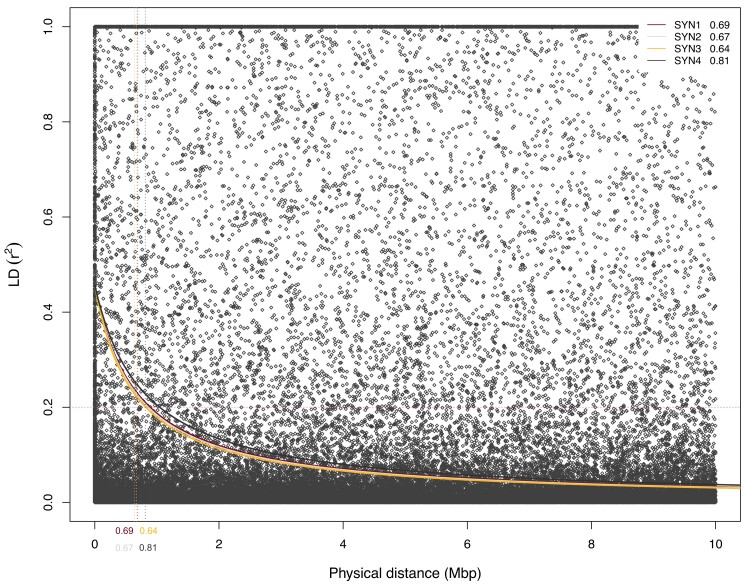
Comparison of LD (*r*^2^) among the 4 different SYN generations of the IWG cultivar MN-Clearwater. Lines fitted to display the distribution are locally weighted smoothing curves. Physical distance is limited to 10 Mbp because the decay distance is <1 Mbp in all populations.

LD (*r*^2^) values were mostly similar among the 4 populations (Fig. 3). The average *r*^2^ value among all 4 populations was 0.093 and ranged from 0.092 (SYN2) to 0.096 (SYN4). The decay of LD at conventional *r*^2^ = 0.2 was the highest for SYN4 generation with decay distance of 0.8 mega base pairs (Mbp) and lowest for SYN3 generation (0.64 Mbp).

### Simulation results for optimal number of parents, trait variance, and heritability

How many parents should be used to initiate IWG SYN populations to obtain and maintain long-term genetic variance and trait performance? The answer to this question provided by simulation analysis revealed that using 20 parents to create a SYN population might result in the best possible outcome (Table 2) as nearly all SYN generations simulated from 20 founder parents led to the highest-observed trait variances. Two exceptions were SYN2 and SYN4 generations initiated from 10 and 25 parents, respectively, where the performances were identical to generations initiated from 20 parents. On the contrary, using 7 parents to create a SYN population led to a reduction in variance in all generations. In the remaining scenarios, the distinction among generations did not follow a consistently obvious pattern. Except for the population initiated with 20 parents, the remaining SYN generations overall performed poorly when advancing to SYN3 and SYN4 generations, albeit this difference was not significant.

**Table 2.** Genotypic (additive, σ^2^_a_) and phenotypic (σ^2^_p_) variances and heritability estimates (*H*^2^) in 4 IWG SYN generations depending on how many parents were used to generate the population. Values in parentheses are standard deviations.

		No. of parents				
Parameter	Generation	7	10	15	20	25
σ^2^_a_	SYN1	0.23 (0.08)	0.26 (0.09)	0.25 (0.08)	0.30 (0.09)	0.25 (0.07)
	SYN2	0.24 (0.08)	0.28 (0.09)	0.25 (0.08)	0.28 (0.09)	0.27 (0.07)
	SYN3	0.23 (0.08)	0.27 (0.09)	0.26 (0.08)	0.28 (0.09)	0.28 (0.07)
	SYN4	0.22 (0.08)	0.26 (0.09)	0.26 (0.08)	0.30 (0.09)	0.27 (0.07)
σ^2^_p_	SYN1	0.35 (0.07)	0.38 (0.07)	0.37 (0.08)	0.40 (0.08)	0.38 (0.06)
	SYN2	0.36 (0.07)	0.38 (0.07)	0.37 (0.08)	0.39 (0.08)	0.38 (0.06)
	SYN3	0.36 (0.07)	0.37 (0.07)	0.39 (0.08)	0.39 (0.08)	0.38 (0.06)
	SYN4	0.34 (0.07)	0.40 (0.07)	0.39 (0.08)	0.41 (0.08)	0.39 (0.06)
*H* ^2^	SYN1	0.66 (0.32)	0.70 (0.28)	0.68 (0.28)	0.75 (0.37)	0.68 (0.22)
	SYN2	0.64 (0.42)	0.77 (0.37)	0.68 (0.25)	0.70 (0.26)	0.74 (0.35)
	SYN3	0.60 (0.14)	0.77 (0.42)	0.67 (0.16)	0.71 (0.32)	0.71 (0.31)
	SYN4	0.63 (0.28)	0.66 (0.09)	0.67 (0.22)	0.72 (0.27)	0.71 (0.29)

The average heritability (*H*^2^) of predicted traits from all generations and number of parents was 0.7, slightly higher compared with the *H*^2^ value supplied to the simulation model (0.6). On average, an insignificant increase in *H*^2^ estimate was observed from SYN1 to SYN2 and reduction when advancing to SYN3 and SYN4. The SYN3 and SYN4 generations also had the lowest estimates despite SYN4 having the estimate closest to the original value (0.68).

**Fig. 4.**
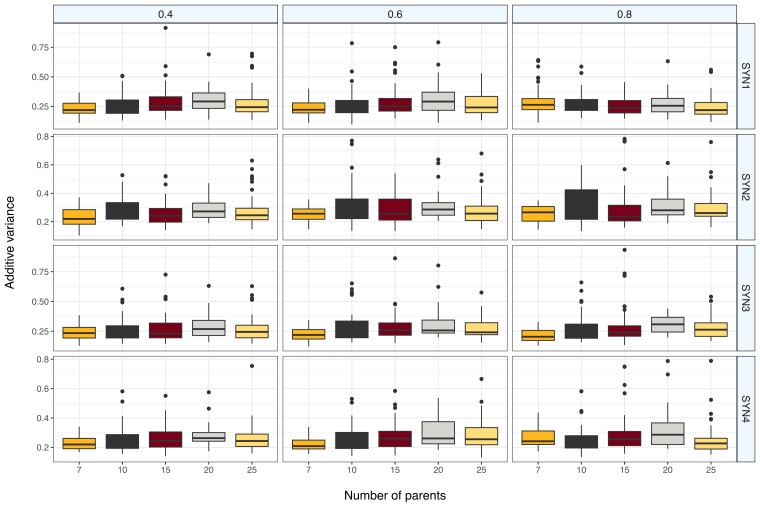
Additive variances (σ^2^_a_) in 4 different IWG SYN generations (rows) for traits with 3 different heritability values, i.e. *H*^2^ = 0.4, 0.6, and 0.8 (columns). Values were simulated in AlphasimR as described in the Materials and Methods section.

Population simulation was further expanded to investigate the effect of varying levels of trait heritabilities, i.e. *H*^2^ = 0.4, 0.6, and 0.8, on σ^2^_a_ in the 4 SYN generations created from different numbers of parents. Across the 3 heritabilities, *H*^2^ of 0.6 had the higher overall variance estimates (Fig. 4). Most SYN4 generations also had lesser variance compared with earlier SYN generations. Similar to the results observed in Table 2, SYNs initiated from 7 parents had less variance in all heritability classes and were significantly different from SYNs generated with 10 or more parents (Supplementary Table 1). In most cases, SYN2 had higher variance than other generations. Across all 4 SYN generations, SYNs initiated from 20 parents had the highest σ^2^_a_ (0.30) followed by populations initiated from 15 parents (0.28). Similarly, the highest σ^2^_a_ among the 4 SYN generations was observed in SYN2 when *H*^2^ = 0.6 and 0.8 (σ^2^_a_ = 0.29 in both cases), followed by SYN3 when *H*^2^ = 0.6 (σ^2^_a_ = 0.28). Despite these observations, the differences for σ^2^_a_ were not significant across the 4 SYN generations (Supplementary Table 2).

### Predicted trait performance

**Fig. 5.**
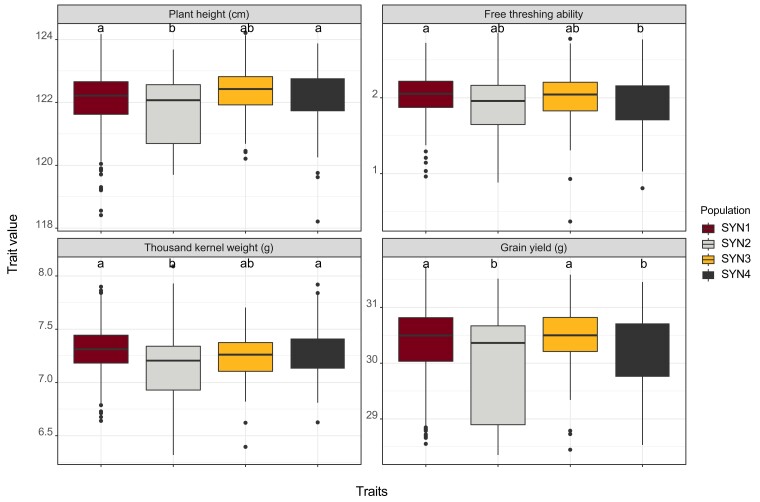
Differences in trait performance of individuals within the 4 SYN generations (SYN1–SYN4) of IWG as predicted by a genomic prediction model. Free threshing ability is measured on a scale of 0–9 and has no units. Common letters above the boxplots indicate that trait means are not significantly different by the Tukey's HSD test at the significance level of *α* = 0.001.

Genomic prediction models were also utilized to compare differences among the 4 SYN generations by predicting trait performances of individuals in each SYN generation. Model predictive abilities ranged from 0.45 to 0.64 (Supplementary Table 3). A few significant differences were observed among the 4 populations for predicted plant height, free threshing ability, seed size measured in terms of thousand kernel weight and grain yield (Fig. 5). Specifically, SYN2 was predicted to have slightly lower height and seed size compared with SYN1 and SYN4; free threshing ability was significantly lower for SYN4 relative to SYN1; grain yield was significantly lower in SYN2 and SYN4 compared with SYN1 and SYN3. Despite a few significantly different scenarios, the HSD at *α* = 0.001 level were only 0.74, 0.09, 0.05, and 0.47 for plant height, free threshing ability, seed size measured as thousand kernel weight, and grain yield, respectively.

## Discussion

In this study, we have described the application of genomic tools and statistical models in cataloging the genetic constitution and variability in IWG SYN populations. In particular, we demonstrate the use of simulation and genomic prediction using real genotyping data to assess the differences among these SYN populations. Our simulation process made the best attempt to mimic the process carried out in the field, i.e. take the population from the previous generation, create random crosses, obtain progeny, and repeat the steps until SYN4 is obtained from SYN1. Based on the results, the following is structured to discuss 3 main topics: (1) genetic differences among 4 SYN generations in IWG, (2) application and limitations of the simulation model, and (3) implications for an applied IWG breeding program.

### Genetic differences among SYN populations

In our evaluations of the 4 SYN generations of the IWG cultivar MN-Clearwater, we observed small and inconsequential genetic differences among them. Measures of diversity indices (Table 1) were near identical among the 4 generations. Despite the LD decay distance being slightly larger in SYN4 (0.8 Mbp), no substantial differences in LD (*r*^2^) values were observed as the mean *r*^2^ for all 4 populations was 0.093. PC analysis placed all but a few individuals of SYN1 and SYN3 generations within a large group. The few SYN1 and SYN3 individuals had no major deviations, i.e. no large differences in missing alleles, homozygosity and heterozygosity content, and minor allele frequencies. We therefore hypothesize that these few individuals are inherently different from others because of genetic drift or mutation, or both.

Genetic gain in a breeding program is mostly determined by the amount of additive genetic variance (σ^2^_a_; Lanzl *et al.* 2023). It is therefore important for plant breeders to estimate σ^2^_a_ accurately and reliably in the selection population. A strong selection pressure, be it phenotypic or genomic selection, could lead to a sharp decline in σ^2^_a_. Availability of abundant σ^2^_a_ is also important for expression of traits in breeding populations (Hill *et al.* 2008). While the SYN populations of IWG do not undergo selection, it is important to maintain high levels of σ^2^_a_ to maintain population performance. As described earlier, the SYN populations of IWG are closed populations that are created from a few parent genotypes. The population size increases each generation from SYN1 to SYN4 as plots are established for the purpose of seed increase to obtain larger amounts of grain for variety release. Despite the increase in population size, there is no external source of genetic variation except drift since the plots are established in isolation from other IWG plots. The total variance is therefore expected to be mostly constant, at least in theory and in the absence of selection and inbreeding, because of the closed population structure (Lande and Barrowclough 1987). Yet the determination of how many parents to use in creating a SYN population and how this affects long-term population variance was not previously explored in IWG. In our results, we observed no apparent reduction in genetic variance as we increase generations from SYN1 to SYN4 because no selection was made in the simulation model.

In terms of field performance, limited empirical data exist in elucidating the differences among different SYN generations of the same SYN variety. In our breeding work, SYN2 and SYN3 generations of MN-Clearwater and a few other experimental candidates have been evaluated in the field for agronomic performance in 2022 and 2023. As we continue such evaluations in coming years by including more candidates in the experiment, we expect to summarize the data in a forthcoming study after collecting enough information in coming field seasons.

### Simulation and trait prediction

Given that field-based seed increase and generation advance are different from the simulation models, there are limitations associated with our simulation methods. In our study, we explored 3 fixed heritability levels, at 0.4, 0.6, and 0.8. This is a simplified assumption as trait heritabilities in IWG vary from less than 0.2 to more than 0.9 (Bajgain *et al.* 2022b). We used these 3 values to broadly accommodate several trait types. The near-constant levels of genetic variance among the 4 SYN populations at all heritability levels do suggest that trait performance in different SYN populations could be similar. To simulate trait architecture in IWG, we used a genome-wide QTL number of 1,000. Many traits in IWG are known to be of complex nature that are controlled by several genes of small effects (Bajgain *et al.* 2022b); some traits such as seed length, seed width, and seed mass are controlled by more than 50 QTL. As more QTL mapping studies are conducted, we expect the number of genome-wide QTL numbers to approach and possibly exceed 1,000.

While the application of real genotypic data to simulate was helpful in recreating the SYN populations, we also acknowledge that the number of crosses we simulated as well as the population size is also smaller than what we expect in the field. The SYN generations at UMN are planted for seed increase and generation advancement in an area approximately 230 m^2^ (2,500 ft^2^). In this plot with a planting rate of 17 kg pure live seed ha^−1^ (15 lb ac^−1^) and survival rate of 80%, approximately 46,000 live plants can be expected. Most IWG plants also tiller profusely and produce many fertile spikes per plant, typically in the range of 50–200. Therefore, the number of crosses in the field easily exceeds the 1,000 crosses we simulated in our model. Likewise, with average seed mass of 7 mg and per plant average seed production of 36 g (data from Cycle 3 breeding population; Bajgain *et al.* 2019), 5,100 seeds can be expected per plant, which is larger than the 100 progeny we parameterized in the model. While these parameters might not perfectly reflect biological populations in the field (e.g. seed production from individual plants), they do allow for a baseline comparison of all SYN populations on a similar scale. As more research studies are conducted and published in IWG, future simulations can benefit from the application of more accurate model parameters.

### Breeding considerations

Parental selection for development of high-performing and stable cultivars is an important aspect of developing cultivars in outcrossing perennial forage and grass crop species (Aastveit and Aastveit 1990; Annicchiarico *et al.* 2016). It is generally understood that σ^2^_a_ values vary between individuals in a large population generated from few to several random crosses. While a single to few recombination events could help improve σ^2^_a_ by disassociating negative and positive covariances between QTL pairs between chromosomes (Lanzl *et al.* 2023), several cycles of recombination events and selection events could be necessary to observe significant genetic progress. In our previous study in IWG, we observed that σ^2^_a_ was the most important in trait expression with dominance having small effects (Bajgain *et al.* 2020a). A breeder of outcrossing crops such as IWG could therefore, in theory, expect larger response in trait expression by using a breeding scheme that utilizes additive genetic variance more efficiently relative to other variance types. This careful selection is even more necessary given the perenniality of IWG as the SYN cultivars need to be productive for multiple cropping seasons. Throughout these multiple seasons, while the environment is changing and the crop is aging, the genetic makeup of the SYN cultivar in a farmer's field is near identical, barring any plant death or germination of volunteer seed. Maximizing σ^2^_a_ is therefore crucial to maintain the productivity of this crop over multiple years.

In grass and forage species with some inbreeding depression, the breeder must choose the number of parents to avoid the effects of inbreeding. As IWG has a severe inbreeding depression (Bajgain *et al.* 2022a), it is important to select and plant genotypically distinct individuals as well as avoid planting clones or similar individuals next to each other. Inbreeding depression may be a risk with too few parents, especially if the parental genotypes are genetically similar. Careful selection of mating parents and crossing design could thus maximize the parent–offspring covariance and improve additive genetic variance (Hill Jr. 1971). Based on the results obtained in this study, we recommend that IWG breeders use at least 10 parents, with 20 being the ideal number, when creating SYN populations in order to maintain abundant genetic variance. There is however yet another logistical consideration regarding the number of parents used to create the SYN population. An increase in the number of parents that need to be excavated from a field (if using field-observed trait performance), cloned, and propagated to establish SYN crossing blocks can add undesired strain on available labor and resources and negatively impact project logistics. Taking these simulation findings and applied breeding experience into consideration, using 25 or more parents might not be ideal.

## Conclusions

In this study, we evaluated variance and trait differences among 4 SYN populations of IWG. Results show that these different populations are genetically quite similar and perform similarly in the field. We observed a reduction in genetic (additive) variance in SYN populations that were created from 7 parents compared with populations that were created using 10 or more parents with 20 parents being the ideal choice. Prediction of a few traits, e.g. plant height, free threshing ability, seed mass, and grain yield among the 4 SYN generations, also showed that the SYN generations are mostly similar with small yet significant differences among them. We expect these results to inform the creation of new SYN cultivars of IWG. The methods used in this study could potentially be used to conduct similar studies in other similar perennial forage and grain crops.

## Supplementary Material

jkae154_Supplementary_Data

## Data Availability

Supplementary File 1 contains genotype data, and Supplementary File 2 contains the R script used for simulation work. All other data necessary for confirming the conclusions of the article are present within the article, figures, and tables. Supplemental material available at G3 online.
