# Supplementary material for: Genetic constitution and variability in synthetic populations of intermediate wheatgrass, an outcrossing perennial grain crop
Source: G3 (Bethesda). 2024 Jul 13;14(9):jkae154. doi: 10.1093/g3journal/jkae154 (PMC11373638; doi:10.1093/g3journal/jkae154)
Supplement: jkae154_Supplementary_Data [file jkae154_supplementary_data.zip › Table_S2_G3-2024-405151.docx]

**Supplemental Table S2:** Tukey’s HSD means and groups for three trait heritabilities across the four synthetic populations. Means followed by a common letter are not significantly different by the Tukey’s HSD test at the significance level of *α* = 0.001.

| *H^2^* | Generation | Value | StD | SE | Min | Max | Q25 | Q50 | Q75 | Group |
| --- | --- | --- | --- | --- | --- | --- | --- | --- | --- | --- |
| 0.4 | SYN1 | 0.27 | 0.11 | 0.01 | 0.13 | 0.91 | 0.20 | 0.24 | 0.30 | a |
|  | SYN2 | 0.26 | 0.09 | 0.01 | 0.13 | 0.63 | 0.20 | 0.24 | 0.30 | a |
|  | SYN3 | 0.27 | 0.10 | 0.01 | 0.13 | 0.73 | 0.20 | 0.24 | 0.30 | a |
|  | SYN4 | 0.26 | 0.08 | 0.01 | 0.14 | 0.75 | 0.20 | 0.24 | 0.30 | a |
| 0.6 | SYN1 | 0.27 | 0.11 | 0.01 | 0.10 | 0.79 | 0.20 | 0.24 | 0.32 | a |
|  | SYN2 | 0.29 | 0.12 | 0.01 | 0.13 | 0.77 | 0.21 | 0.25 | 0.33 | a |
|  | SYN3 | 0.28 | 0.11 | 0.01 | 0.14 | 0.86 | 0.21 | 0.24 | 0.32 | a |
|  | SYN4 | 0.26 | 0.10 | 0.01 | 0.13 | 0.90 | 0.20 | 0.23 | 0.31 | a |
| 0.8 | SYN1 | 0.27 | 0.09 | 0.01 | 0.11 | 0.64 | 0.20 | 0.25 | 0.31 | a |
|  | SYN2 | 0.29 | 0.12 | 0.01 | 0.13 | 0.78 | 0.21 | 0.25 | 0.32 | a |
|  | SYN3 | 0.27 | 0.10 | 0.01 | 0.13 | 0.93 | 0.20 | 0.24 | 0.31 | a |
|  | SYN4 | 0.27 | 0.13 | 0.01 | 0.13 | 0.88 | 0.20 | 0.23 | 0.30 | a |
