# Supplementary material for: Genetic constitution and variability in synthetic populations of intermediate wheatgrass, an outcrossing perennial grain crop
Source: G3 (Bethesda). 2024 Jul 13;14(9):jkae154. doi: 10.1093/g3journal/jkae154 (PMC11373638; doi:10.1093/g3journal/jkae154)
Supplement: jkae154_Supplementary_Data [file jkae154_supplementary_data.zip › Table_S3_G3-2024-405151.docx]

**Supplemental Table S3:** Predictive abilities, i.e. correlations between model-predicted trait value and field-observed trait values, for the traits plant height, free threshing ability, thousand kernel weight, and grain yield as obtained from the rrBLUP package.

| **Plant height** | **Free threshing ability** | **Thousand kernel weight** | **Grain yield** |
| --- | --- | --- | --- |
| 0.534552 | 0.607208 | 0.633263 | 0.432018 |
| 0.517369 | 0.605631 | 0.636752 | 0.489023 |
| 0.509542 | 0.585255 | 0.668377 | 0.514813 |
| 0.558282 | 0.60824 | 0.606382 | 0.51565 |
| 0.414356 | 0.551665 | 0.567368 | 0.418805 |
| 0.420892 | 0.585141 | 0.612444 | 0.448304 |
| 0.505933 | 0.581747 | 0.594162 | 0.344132 |
| 0.451865 | 0.586127 | 0.566399 | 0.450955 |
| 0.569577 | 0.566292 | 0.649027 | 0.462777 |
| 0.481241 | 0.621407 | 0.626927 | 0.495177 |
| 0.540395 | 0.54713 | 0.606555 | 0.53797 |
| 0.486989 | 0.575606 | 0.664967 | 0.459938 |
| 0.488123 | 0.59267 | 0.655455 | 0.4504 |
| 0.495805 | 0.536951 | 0.57165 | 0.489327 |
| 0.475349 | 0.582078 | 0.663094 | 0.453844 |
| 0.48413 | 0.648883 | 0.672947 | 0.459186 |
| 0.559875 | 0.622625 | 0.643074 | 0.355363 |
| 0.46205 | 0.622009 | 0.626391 | 0.424896 |
| 0.480895 | 0.645738 | 0.632644 | 0.478445 |
| 0.482406 | 0.64744 | 0.655191 | 0.435575 |
| 0.567134 | 0.600005 | 0.626078 | 0.434195 |
| 0.534534 | 0.599845 | 0.609999 | 0.40727 |
| 0.556493 | 0.640368 | 0.68005 | 0.482726 |
| 0.569183 | 0.663198 | 0.617179 | 0.411748 |
| 0.557677 | 0.646253 | 0.633305 | 0.474015 |
| 0.456158 | 0.614234 | 0.667964 | 0.459198 |
| 0.514706 | 0.546923 | 0.693662 | 0.52043 |
| 0.545864 | 0.627488 | 0.581691 | 0.438725 |
| 0.474603 | 0.549615 | 0.614395 | 0.402302 |
| 0.567489 | 0.570065 | 0.654121 | 0.423213 |
| 0.506074 | 0.653586 | 0.606777 | 0.463052 |
| 0.533627 | 0.605843 | 0.60072 | 0.462648 |
| 0.51071 | 0.57174 | 0.675111 | 0.447793 |
| 0.484534 | 0.676071 | 0.626884 | 0.429438 |
| 0.418006 | 0.560253 | 0.67378 | 0.395405 |
| 0.479707 | 0.643633 | 0.682952 | 0.493227 |
| 0.470009 | 0.639126 | 0.615639 | 0.386918 |
| 0.511417 | 0.615253 | 0.634698 | 0.4796 |
| 0.523489 | 0.587271 | 0.621255 | 0.444115 |
| 0.401888 | 0.602319 | 0.681507 | 0.422561 |
| 0.503651 | 0.563502 | 0.621506 | 0.494402 |
| 0.478771 | 0.567843 | 0.632549 | 0.452937 |
| 0.522613 | 0.704796 | 0.598386 | 0.449563 |
| 0.500583 | 0.608029 | 0.65452 | 0.446167 |
| 0.528248 | 0.669821 | 0.610686 | 0.392583 |
| 0.523656 | 0.613362 | 0.598808 | 0.517419 |
| 0.536099 | 0.62532 | 0.72738 | 0.494552 |
| 0.519187 | 0.575368 | 0.694627 | 0.487926 |
| 0.492903 | 0.581092 | 0.599504 | 0.494538 |
| 0.415617 | 0.586011 | 0.617548 | 0.373785 |
| 0.472655 | 0.557128 | 0.68523 | 0.407777 |
| 0.499806 | 0.609046 | 0.605949 | 0.439391 |
| 0.485 | 0.652533 | 0.658905 | 0.454613 |
| 0.494242 | 0.585045 | 0.678851 | 0.463135 |
| 0.484763 | 0.645502 | 0.615825 | 0.462985 |
| 0.551443 | 0.669185 | 0.663709 | 0.455904 |
| 0.475783 | 0.606386 | 0.623951 | 0.442168 |
| 0.537474 | 0.625822 | 0.630012 | 0.456494 |
| 0.434734 | 0.569235 | 0.685042 | 0.482845 |
| 0.519458 | 0.588623 | 0.620631 | 0.45509 |
| 0.549095 | 0.61475 | 0.600749 | 0.3794 |
| 0.447194 | 0.645982 | 0.599718 | 0.472304 |
| 0.523027 | 0.591626 | 0.615636 | 0.448999 |
| 0.420603 | 0.529368 | 0.677533 | 0.399433 |
| 0.505968 | 0.644693 | 0.62884 | 0.439606 |
| 0.440808 | 0.583461 | 0.652514 | 0.386278 |
| 0.467433 | 0.594907 | 0.713223 | 0.47552 |
| 0.546177 | 0.57312 | 0.649583 | 0.420383 |
| 0.536436 | 0.587313 | 0.590058 | 0.469742 |
| 0.455602 | 0.626435 | 0.638295 | 0.44923 |
| 0.505628 | 0.531838 | 0.686164 | 0.388308 |
| 0.528909 | 0.607652 | 0.608227 | 0.428701 |
| 0.494175 | 0.636824 | 0.644183 | 0.516238 |
| 0.497234 | 0.622209 | 0.669974 | 0.392005 |
| 0.517287 | 0.634434 | 0.584603 | 0.414861 |
| 0.498105 | 0.701353 | 0.667603 | 0.354421 |
| 0.499006 | 0.602832 | 0.632151 | 0.489097 |
| 0.532564 | 0.670958 | 0.631335 | 0.517267 |
| 0.484661 | 0.675356 | 0.658416 | 0.408242 |
| 0.442885 | 0.700192 | 0.619932 | 0.502297 |
| 0.452127 | 0.6004 | 0.659422 | 0.440786 |
| 0.492569 | 0.582155 | 0.649242 | 0.445975 |
| 0.53951 | 0.599117 | 0.682785 | 0.414491 |
| 0.414195 | 0.629047 | 0.65018 | 0.438025 |
| 0.50923 | 0.600736 | 0.611188 | 0.432061 |
| 0.516578 | 0.612761 | 0.609288 | 0.501097 |
| 0.515393 | 0.665433 | 0.686278 | 0.450103 |
| 0.480739 | 0.632308 | 0.592172 | 0.490102 |
| 0.480464 | 0.540355 | 0.636107 | 0.454296 |
| 0.569748 | 0.521894 | 0.618239 | 0.494574 |
| 0.481013 | 0.602461 | 0.667113 | 0.404074 |
| 0.489327 | 0.629043 | 0.685795 | 0.515962 |
| 0.556313 | 0.604941 | 0.601189 | 0.430355 |
| 0.5764 | 0.576218 | 0.629674 | 0.465153 |
| 0.563444 | 0.671637 | 0.691065 | 0.538825 |
| 0.552963 | 0.679404 | 0.65094 | 0.439244 |
| 0.486768 | 0.631282 | 0.615687 | 0.500652 |
| 0.619713 | 0.571632 | 0.652687 | 0.451167 |
| 0.561964 | 0.590851 | 0.619375 | 0.425706 |
| 0.52814 | 0.650691 | 0.687331 | 0.429955 |
